# Antibacterial Polymers Based on Poly(2-hydroxyethyl methacrylate) and Thiazolium Groups with Hydrolytically Labile Linkages Leading to Inactive and Low Cytotoxic Compounds

**DOI:** 10.3390/ma14237477

**Published:** 2021-12-06

**Authors:** Rocío Cuervo-Rodríguez, Fátima López-Fabal, Alexandra Muñoz-Bonilla, Marta Fernández-García

**Affiliations:** 1 Facultad de Ciencias Químicas, Universidad Complutense de Madrid, Avenida Complutense s/n, Ciudad Universitaria, 28040 Madrid, Spain; rociocr@quim.ucm.es; 2 Hospital Universitario de Móstoles, C/Dr. Luis Montes s/n, 28935 Móstoles, Spain; flopezf@salud.madrid.org; 3 Instituto de Ciencia y Tecnología de Polímeros (ICTP-CSIC), C/Juan de la Cierva 3, 28006 Madrid, Spain

**Keywords:** antibacterial polymers, resistant bacteria, RAFT polymerization, degradable polymers, pH-sensitive linkage

## Abstract

Herein, we develop a well-defined antibacterial polymer based on poly(2-hydroxyethyl methacrylate) (PHEMA) and a derivative of vitamin B1, easily degradable into inactive and biocompatible compounds. Hence, thiazole moiety was attached to HEMA monomer through a carbonate pH-sensitive linkage and the resulting monomer was polymerized via reversible addition-fragmentation chain transfer (RAFT) polymerization. *N*-alkylation reaction of the thiazole groups leads to cationic polymer with thiazolium groups. This polymer exhibits excellent antibacterial activity against methicillin-resistant *Staphylococcus aureus* (MRSA) with an MIC value of 78 µg mL^−1^, whereas its degradation product, thiazolium small molecule, was found to be inactive. Hemotoxicity studies confirm the negligible cytotoxicity of the degradation product in comparison with the original antibacterial polymer. The degradation of the polymer at physiological pH was found to be progressive and slow, thus the cationic polymer is expected to maintain its antibacterial characteristics at physiological conditions for a relative long period of time before its degradation. This degradation minimizes antimicrobial pollution in the environment and side effects in the body after eradicating bacterial infection.

## 1. Introduction

In recent years, the development of new antibacterial materials has attracted increasing attention as antibiotic resistant infections are becoming one of the most healthcare challenges facing the world [1]. The overuse of antibiotics by humans, animals and in agriculture is considered one of the main causes of antimicrobial resistance development (AMR). This antibiotic consumption is continuously growing and is responsible for the increased antibiotic pollution in the environment [2]. Antibiotics in sub-lethal levels found in soil, rivers, lakes, drinking water, etc., can promote the presence of antibiotic-resistant genes (ARGs) in the environment contributing to bacterial resistance to antibiotics [3,4]. Then, these environmental concerns also have to be taken into consideration in the design of new antibacterial agents in order to reduce antibiotics pollution [5,6,7,8]. In the search of new antimicrobial agents, antibacterial polymers have emerged as very potent alternatives due to their excellent effectivities and low tendency to generate resistance [9,10,11,12]. Additionally, their outstanding physico-chemical properties made them good candidates to fabricate antimicrobial materials for applications such as food packaging, biomaterials and medical devices [13,14,15,16]. In the few last years, a large number of new synthetic polymers as well as modification of existing polymers by incorporation of active groups have been developed with very potent activity [12,17,18,19]. These synthetic antimicrobial polymers are also studied in combination therapy with other agents such as nitric oxide, antibiotics, and metal nanoparticles to increase its effectivity even more [20,21].

Typical antimicrobial polymers stand out for their chemical stability; however, in the last few years, self-degradable and biodegradable polymer systems are also needed to reduce not only antimicrobial pollution in the environment but also degradability in vivo minimizes side effects in the body after eradicating bacterial infection [22,23,24]. The biodegradability of the polymers can be achieved by the presence or incorporation of labile chemical linkages such as ester, amide and carbonate bonds. Biodegradable polyesters including polylactic acid (PLA), polysaccharides and polyitaconate derivatives have been chemically modified to add antimicrobial moieties in their structures [25,26,27,28,29]. Other strategies involve the incorporation of degradable linkages in the structure of antimicrobial polymers. For instance, imidazolium antimicrobial polymers that incorporate pH-dependent degradable links (including carbonate, hemiaminal, ester and urea) are able to degrade at basic pH, reducing the risk of secondary contamination and resistance development after treatment [30,31].

**Scheme 1:**
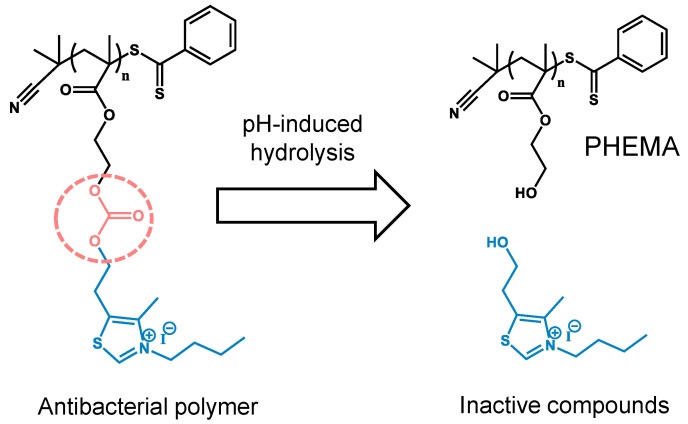
Representation of antibacterial polymers containing pH-sensitive linkers.

In this paper, we report degradability studies of antimicrobial polymers based on poly(2-hydroxyethyl methacrylate) (PHEMA) [32] with hydrolytically labile carbonate ester side chain as pH-degradable linkage of thiazolium antimicrobial groups (a derivative of Vitamin B1) (Scheme 1). By this approach, antimicrobial polymers with excellent effectivity against bacteria, including resistance strains, are able to degrade under neutral or basic media leading biocompatible and non-cytotoxic components. Hydrolysis of the carbonate esters affords PHEMA, which is a well-known polymer with excellent biocompatibility, cytocompatibility and minimal immunogenicity, which is widely used as biomaterials for different biomedical applications [33]. In addition to PHEMA, small thiazolium units are also delivered to the medium, showing almost negligible cytotoxicity and low antibacterial effectivity.

## 2. Materials and Methods

### 2.1. Materials

The methacrylic monomer 2-(((2-(4-methylthiazol-5-yl)ethoxy)carbonyl)oxy)ethyl methacrylate (MTZ) was prepared according to the procedure described previously [32]. Anhydrous *N,N*-dimethylformamide (DMF, 99.8%), anhydrous tetrahydrofuran (THF, 99.9%), dimethylsulfoxide (DMSO, 99%), n-hexane (≥95%), 5-(2-hydroxyethyl)-4-methylthiazole (98%), 1-iodobutane (99%) and RAFT agent 2-cyano-2-propyl benzodithioate (CPBD, >97%), were purchased from Sigma Aldrich and used as received. 2,2′-Azobisisobutyronitrile (AIBN, 98%, Acros) was recrystallized twice from methanol. Cellulose dialysis membranes (CelluSep T-series and H1) were acquired from Membrane Filtration Products, Inc. Phosphate buffered saline powder (pH 7.4) was acquired from Sigma-Aldrich, buffer solution at pH 9 was obtained from Chem Lab.

For biological studies, sodium chloride solution (NaCl suitable for cell culture, BioXtra), Triton X-114 (laboratory grade) were obtained from Sigma-Aldrich. Growth medium, BBL Mueller–Hinton broth, was purchased from Becton Dickinson Company and Columbia agar (5% sheep blood) plates from BioMérieux. *Staphylococcus aureus* resistant to methicillin and oxacillin (*S. aureus*, ATCC 43300) used as bacterial strain was purchased from Oxoid, Thermo Fisher Scientific.

### 2.2. N-Alkylation Reaction of 5-(2-hydroxyethyl)-4-methylthiazole (TZ-Bu)

**Scheme 2:**
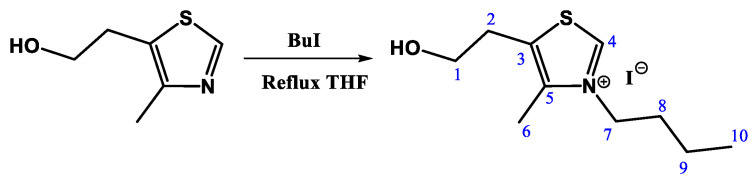
*N*-alkylation reaction of 5-(2-hydroxyethyl)-4-methylthiazole. Synthesis of TZ-Bu.

Briefly, 5-(2-hydroxyethyl)-4-methylthiazole (0.5 g, 3.5 mmol) was dissolved in 5.5 mL of anhydrous THF (Scheme 2). Subsequently, 1.4 mL of 1-iodobutane (122 mmol) was added to the solution and the reaction was heated under reflux in an inert atmosphere. The progress of the reaction was followed by NMR spectroscopy. After the reaction was completed (7 days), the mixture was cooled added to a large excess of hexane under stirring. The solvent was removed by decantation and the product (brown oil) was washed repeatedly with hexane and ethyl ether and dried under vacuum (yield: 65%).

^1^H NMR (300 MHz, DMSO-d6) δ (ppm): 10.01 (s; H-4; 1H), 5.19 (t; J = 5.0 Hz; OH; 1H), 4.52–4.40 (m; H-7; 2H), 3.71–3.59 (m; H-1; 2H), 3.03 (t; J = 5.6 Hz; H-2; 2H), 2.48 (s; H-6; 3H), 1.87–1.71 (m; H-8, 2H), 1.34 (dq; J = 7.4 Hz; H-9; 2H), 0.94 (t; J = 7.3 Hz; H-10; 3H).

^13^C NMR (75 MHz, DMSO-d6) δ (ppm): 156.04 (C-4), 141.65 (C-5), 135.59 (C-3), 59.77 (C-1), 52.66 (C-7), 30.91 (C-8), 29.58 (C-2), 19.00 (C-9), 13.43 (C-6), 11.30(C-10).

### 2.3. N-Alkylation Reaction of 2-(((2-(4-Methylthiazol-5-yl)ethoxy)carbonyl)oxy)ethyl Methacrylate. Synthesis of MTZ-Bu

**Scheme 3:**
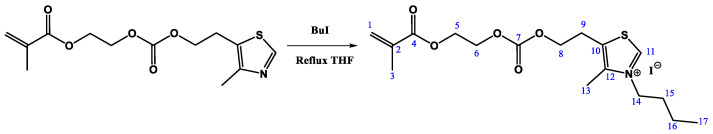
*N*-alkylation reaction of 2-(((2-(4-methylthiazol-5-yl)ethoxy)carbonyl)oxy)ethyl methacrylate. Synthesis of MTZ-Bu.

Cationic monomer MTZ-Bu was synthesized by *N*-alkylation reaction of the thiazol group with 1-iodobutane (Scheme 3). In a glass tube, the monomer MTZ (0.6 g, 20.0 mmol) was dissolved in 1.5 mL of anhydrous THF. Then, a great excess of 1-iodobutane (2.3 mL, 20.0 mmol) was added to the solution and the reaction was heated under reflux for 7 days under inert atmosphere. The reaction was followed by NMR spectroscopy. Then, a large excess of hexane was added to the reaction mixture under stirring to remove the unreacted reagent. The solvents were removed by decantation and the product (dark orange oil) was further dried under vacuum (yield: 61%).

^1^H NMR (300 MHz, DMSO-d6) δ (ppm): 10.06 (s; H-11; 1H), 6.02 (dq; J = 1.9, 1.0 Hz; H-1; 1H), 5.71 (quint; J = 1.6 Hz; H-1; 1H), 4.51–4.40 (m; H-14; 2H), 4.37–4.25 (m; H-5, H-6 and H-8; 6H), 3.30 (t; H-9; 2H), 2.48 (s; H-13; 3H), 1.87 (dd; J = 1.6, 1.0 Hz; H-3; 3H), 1.83–1.70 (m; H-15; 2H), 1.32 (dq; J = 14.6, 7.3 Hz; H-16; 2H), 0.92 (t; J = 7.4 Hz; H-17; 3H).

^13^C NMR (75 MHz, DMSO-d6) δ (ppm): 166.29 (C-4), 156.63 (C-7), 154.08 (C-11), 143.85 (C-12), 135.50 (C-10), 133.54 (C-2), 126.20 (C-1), 66.53 (C-8), 65.69 (C-6), 62.28 (C-5), 52.75 (C-14), 30.80 (C-15), 25.66 (C-9), 18.86 (C-3), 17.91 (C-16), 13.32 (C-13), 11.22 (C-17).

### 2.4. Polymerization via RAFT of MTZ. Synthesis of the Polymer PMTZ-Bu

**Scheme 4:**
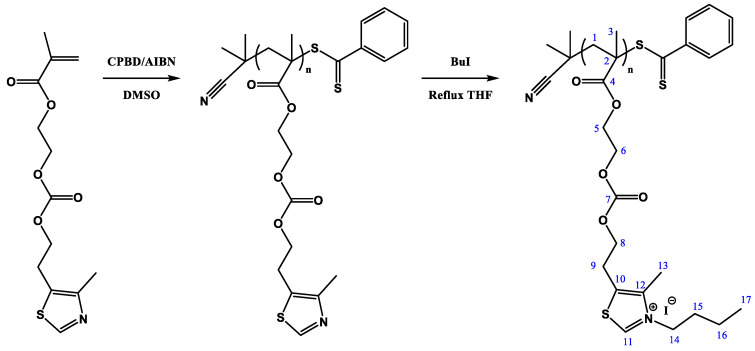
Synthesis of the cationic polymer PMTZ-Bu.

In this process, the monomer MTZ (1.495, 5 mmol), RAFT agent CPBD (0.044 g, 0.2 mmol) and the initiator AIBN (0.016 g, 0.1 mmol) were dissolved in 5 mL of DMSO at a total concentration of 1 M (Scheme 4). The mixture was deoxygenated by purging argon for 15 min and then was placed in an oil bath at 70 °C during 6.5 h under stirring. Afterwards, the reaction was cooled down to ambient temperature and the resulting polymer was purified by dialysis against water (molecular weight cut-off, MWCO 3500 Da). The polymer was labelled as PMTZ (1.32 g, yield: 88%, conversion determined by NMR: 94%). Then, cationic polymer, PMTZ-Bu was obtained by *N*-alkylation with 1-iodobutane. The polymer PMTZ (0.7 g) was dissolved in 6 mL of anhydrous DMF, and a large excess of 1-iodobutane was subsequently added (1 mL). The mixture was deoxygenated under stirring and heated under reflux until a high degree of modification was achieved. The progress of the reaction was followed by NMR spectroscopy. The resulting cationic PMTZ-Bu polymer was purified by precipitation into n-hexane followed by dialysis against distilled water and finally was isolated by freeze-drying. The degree of quaternization analyzed by NMR was almost quantitative.

^1^H NMR (700 MHz, DMSO-d6) δ (ppm): 10.15 (bs; H-11;1H), 4.5 (s; H-14; 2H), 4.30 (bs; H-6 and H-8; 4H), 4.14 (bs; H-5; 2H), 3.33 (bs; H-9; 2H), 2.45 (bs; H-13; 3H), 1.80 (bs; H-15; 2H), 1.9–1.57 (m; H-1; 2H), 1.34 (s; H-16; 2H), 1.10–0.60 (m; H-3; 3H), 0.92 (s; H-17; 3H).

^13^C NMR (176 MHz, DMSO-d6) δ (ppm): 177.27 (C-4); 157.17 (C-7), 154.48 (C-11), 143.44 (C-12), 133.75 (C-10), 67.08 (C-8), 65.73 (C-6), 63.04 (C-5), 53.67 (C-1), 53.67 (C-14), 44.67 (C-2), 31.34 (C-15), 26.20 (C-9), 19.43 (C-16), 18.50 (C-3), 13.90 (C-13), 11.96 (C-17).

### 2.5. Characterization

^1^H and ^13^C NMR spectra were recorded on Bruker DPX spectrometers (300 MHz and 700 MHz) at room temperature using as solvents CDCl3 (99.8% D, Sigma-Aldrich), DMSO-d6 99.8% D, VWR Chemicals).

### 2.6. Evaluation of Monomer and Polymer Hydrolysis

The monomer and polymer degradation kinetics were determined by ^1^H NMR. Briefly, 200 mg of the sample (MTZ-Bu or PMTZ-Bu) were dissolved in 1 mL of DMSO and then 19 mL of the corresponding buffer was added dropwise. The hydrolysis reactions were carried out at 37 °C in an oil bath. At various time points, samples (1 mL) were drawn from the mixture and frozen to remove the solvent under vacuum. Subsequently, the samples were re-dissolved in deuterated DMSO-d6 and analyzed by ^1^H NMR. To obtain a good signal-to-noise ratio at these concentrations, 128 scans were performed.

### 2.7. Antibacterial Activity Measurements

The antibacterial activity of the cationic polymer PMTZ-Bu and the corresponding degradation product, TZ-Bu, was tested by measuring the minimum inhibition concentrations (MICs) following a standard broth dilution method according to the Clinical Laboratory Standards Institute (CLSI) [34]. *S. aureus* bacteria were grown on 5% sheep blood Columbia agar plates during 24 h at 37 °C. Subsample of this culture was diluted to give a concentration of 10^6^ colony-forming units (CFU) mL^−1^ in fresh Mueller–Hinton broth. Tested samples (PMTZ-Bu and TZ-Bu) were dissolved in Mueller–Hinton broth medium at a concentration of 5 mg mL^−1^ and 100 μL from these solutions were placed in the first column of a 96-well round-bottom microplate. Then, 50 μL of broth was added into the rest of the wells and serial two-fold dilutions were performed. From the first column, polymer solution (50 μL) was diluted by 2-fold serial dilutions in the rest of the wells followed by the addition of 50 μL of the bacterial to yield a total volume of 100 μL and a bacterial concentration of ca. 5 × 10^5^ CFU mL^−1^. A positive control without polymer and a negative control without bacteria were also performed. The plates were incubated at 37 °C during 24 h, and the MIC values were determined by checking the absence of bacterial growth visually. All the tests were performed in triplicate.

### 2.8. Hemolysis Assay

Hemolysis studies were carried out to evaluate the cytotoxicity of the cationic polymer and of the degradation product as previously described [26,35]. Fresh human blood was collected from healthy donors directly into blood collecting tubes containing EDTA to prevent coagulation. The tubes were centrifuged at 3500 rpm during 20 min and the red blood cells (RBCs) at the bottom were collected and washed several times with sterile PBS. Subsequently, RBCs were diluted with PBS to give a stock suspension of 5% (*v*/*v*). Solutions of PMTZ-Bu and TZ-Bu in PBS were prepared at a concentration of 40,000 μg mL^−1^. Then, these polymer solutions were mixed with the RBC suspension in serial two-fold dilution in 96-well round-bottom microplates. Similarly, Triton X-114 (1% *v/v* solution in PBS) was used as positive control for 100% of hemolysis while PBS was used as negative control for 0% hemolysis. The microplates were incubated for 1 h at 37 °C, and then, were centrifuged at 1000 rpm for 10 min. The supernatant in each well was transferred to new microplates. Hemolytic activity was analyzed as a function of the released hemoglobin by measuring the absorbance at 550 nm using a microplate reader (Synergy HTX Multi-Mode Reader spectrophotometer, Bio-Tek). Absolute achromatic supernatant solution means no hemolysis (PBS solution, *A_negative control_*) whereas red solution indicates complete hemolysis (Triton X-114, *A_positive control_*). The percentage of hemolysis was calculated as follows:

$$\%\;Hemolysis\;=\;\frac{A_{sample}-A_{negative\;control}}{A_{positive\;control}-A_{negative\;control}}\;\times \;100$$

The data were expressed as mean ± SD (n = 3) of three experiments.

## 3. Results and Discussion

### 3.1. Material Design and Synthesis

In the design of the antibacterial polymer based on biocompatible PHEMA, cationic thiazolium groups derived from vitamin B was incorporated within the structure through a pH-labile carbonate ester linkage (see Scheme 4). In a first step, the MTZ functional monomer, containing the thiazole group and the carbonate link, was synthesized as previously described by our group [32]. Then, the corresponding antibacterial polymer was synthesized via RAFT controlled polymerization of MTZ monomer. It was polymerized at 65 °C in DMSO in the presence of CPBD RAFT agent, with AIBN as the radical initiator at a ratio of [MTZ]:[CPBD]:[AIBN] = 50:2:1. High conversion, 94%, was obtained with a high level of control over the polymerization process. The molecular weight was found to be 10,400 and 12,900, estimated by NMR and by SEC, respectively; while the polydispersity index was 1.15, determined by SEC, indicating good control over the polymerization reaction. Subsequently, alkylation at the nitrogen atom of the thiazole ring was performed with 1-iodobutane leading the corresponding cationic polymer PMTZ-Bu. In addition, the monomer MTZ as well as the precursor 5-(2-hydroxyethyl)-4-methylthiazole were subjected to *N*-alkylation with 1-iodobutane. The resulting cationic monomer, MTZ-Bu, was employed to study the hydrolysis of the carbonate group in comparison with the polymer PMTZ-Bu, due to its low molecular weight this analysis would result easier. On the other hand, the cationic precursor TZ-Bu, which is one of the degradation products, was synthesized in order to determine its antimicrobial activity and hemotoxicity, and to verify its formation by monomer and polymer hydrolysis. In all the cases, a high degree of modification, almost quantitative, was achieved.

### 3.2. pH-Induced Hydrolysis Profiles

**Figure 1:**
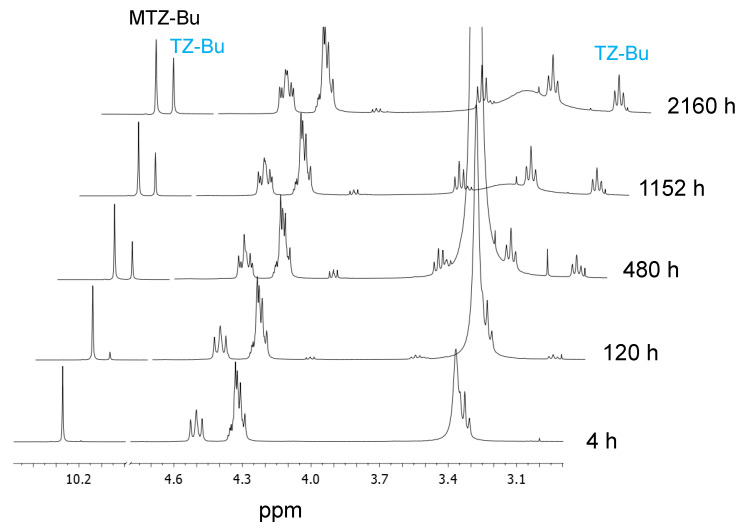
^1^H-NMR spectra of the MTZ-Bu at different percentages of hydrolysis.

**Figure 2:**
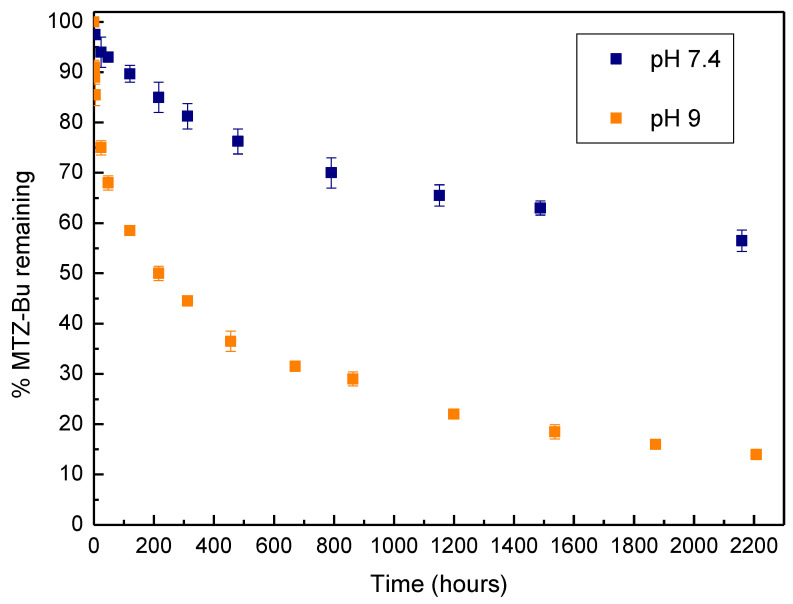
Degradation profile of the monomer MTZ-Bu at pH 7.4 and 9.

Before analyzing the hydrolysis kinetic of the cationic polymer, the monomer MTZ-Bu was firstly studied, as a low molecular weight compound can be measured easily by ^1^H NMR spectroscopy. The hydrolysis kinetic was studied at 37 °C and pH 7.4, representative of the physiological conditions, and also at pH 9 to accelerate the hydrolysis rate of the carbonate ester, as described in the experimental section [36]. The hydrolysis of the carbonate ester moieties leads to the formation of HEMA and 3-butyl-5-(2-hydroxyethyl)-4-methylthiazol-3-ium iodide (TZ-Bu), and the liberation of CO_2_ molecule. Figure 1 displays the ^1^H NMR spectra obtained during the hydrolysis reaction, clearly illustrating the reduction in the intensity of the signals associated to the thiazolium moieties attached to the polymers (e.g., 10.2 ppm attributed to the proton of thiazolium ring, H-11, Scheme 3), and the simultaneous appearance of the signals of the degradation products (e.g., ~10.1 ppm attributed to the proton of thiazolium ring, H-4, Scheme 2). So, hydrolysis percentage of the carbonate moieties was calculated from the ^1^H NMR spectra of the samples withdrawn at specific time, due to the decrease in the signal intensity of the methine proton of the thiazolium ring (δ ~10.2 ppm) in the MTZ-Bu and the increase in the signal intensity of the methine proton of this ring (δ ~10.1 ppm) in the degradation product (Tz-Bu). The percentage of hydrolysis was also performed using other signals of the spectra, such as the signal intensity increment of the methylene protons adjacent to the thiazolium ring (δ ~3.00 ppm) corresponding to the alcohol product (Tz-Bu) (see Figure 1). The final hydrolysis percentage was expressed as the mean value of different calculations ± standard deviation (Figure 2). Hydrolysis of the carbonate group occurs continuously and sustained over time.

As depicted in Figure 2, MTZ-Bu undergoes hydrolysis under the applied conditions. The carbonate ester groups gradually degrade under each studied pH. The hydrolysis rate is strongly affected by the pH of the medium, being much higher at alkaline condition (pH = 9) than at physiological conditions. While in alkaline buffer at 37 °C, hydrolysis of 50% requires only 216 h, and at pH 7.4, 2160 h are needed to degrade 50% of MTZ-Bu in PBS. The rapid hydrolysis under alkaline conditions is in a good agreement with the previously reported results [30,31,36].

**Figure 3:**
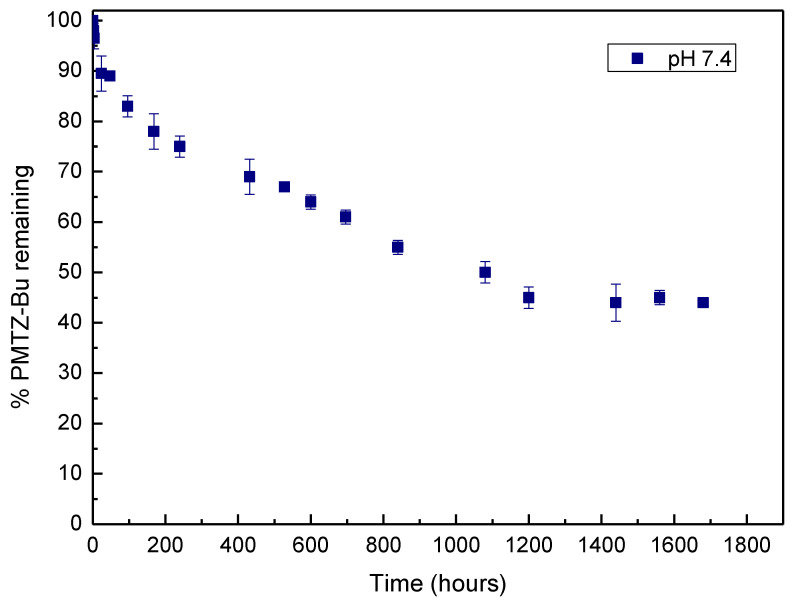
Degradation profile of the antibacterial polymer PMTZ-Bu at pH 7.4.

Subsequently, we further studied the hydrolysis of the antibacterial polymer PMTZ-Bu by incubating the polymer in PBS buffer at physiological conditions (pH = 7.4, 37 °C) with intermittent sampling and subsequent ^1^H NMR spectroscopic analysis to measure the hydrolysis kinetics. In accordance with the hydrolysis rate of the monomer at physiological conditions, a slow degradation rate at neutral pH is expected leading to PHEMA and TZ-Bu as degradation products. Percentage degradation at each period of time was calculated from the disappearance of signal from the methine proton of the thiazolium ring (-CH-, broad singlet around 10.5 ppm) corresponding to the intact polymer and the appearance of signal of the methine proton (singlet, δ ~10.1 ppm), owing to the degradation product TZ-Bu. Figure 3 displays the degradation profile of the hydrolysis behavior of the polymer at physiological conditions. The rate of hydrolysis was quite similar to that cationic monomer MTZ-Bu, although slightly faster. Therefore, the cationic polymer is expected to maintain its antibacterial characteristics at physiological conditions for a relatively long period of time before its degradation.

### 3.3. Antibacterial and Hemolytic Activity

The in vitro antibacterial activity of the cationic polymer PMTZ-Bu and its degradation product TZ-Bu was evaluated against *S. aureus* resistant to methicillin and oxacillin. While the polymer shows very good inhibitory activity against the resistant bacteria, with a MIC value of 78 µg mL^−1^, the hydrolysis of the carbonate link leads to a less active product, TZ-Bu, as reflected in the MIC value at 1250 µg mL^−1^, which is over 16 times the concentration of its polymeric precursors. 

**Figure 4:**
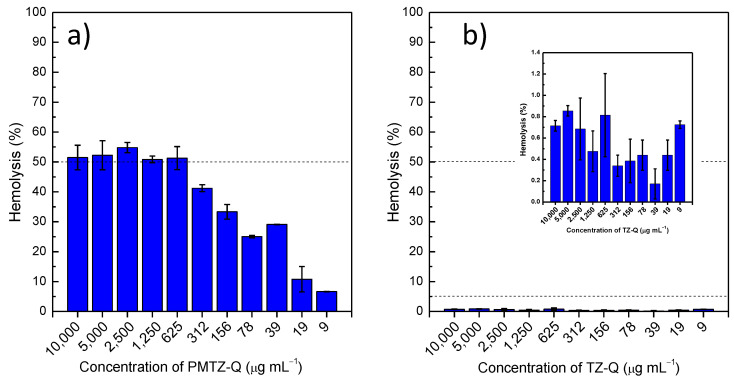
Hemolysis assays for (**a**) the cationic antibacterial polymer PMTZ-Bu and (**b**) for its degradation product TZ-Bu (inset, a magnification of the graph).

The non-toxicity and biocompatibility of the antimicrobial materials is also crucial for biomedical applications. In this sense, hemolysis assay is a common study used to evaluate the cytotoxicity of the antimicrobial compounds. In this work, hemolytic activity of the cationic antibacterial polymer PMTZ-Bu and its degradation product TZ-Bu was tested with human red blood cells. As shown in Figure 4a, the cationic polymer PMTZ-Bu caused more than 50% of hemoglobin leakage from RBCs at a concentration above 625 µg mL^−1^, which is stablished as the HC_50_ value [37,38]. Additionally, the selectivity value for bacterial cells over mammalian cells estimated by the ratio of HC_50_ to MIC values, was found to be HC_50_/MIC = 8. This value is comparable with other antimicrobial compounds such as MSI-78, a derivative of the natural host-defense peptide magainin, with a selectivity of ~16 for MRSA strain [39]. However, this value is far from other antimicrobial polymers designed to have low cytotoxicity, for instance using biobased derivatives, by controlling hydrophobic/hydrophilic balance, incorporating PEG or by glycosylation [17,26,35,40]. In contrast, in Figure 4b no hemolysis was observed (values well-below 5%) for the low molecular weight degradation product within the tested concentration range (up to 10 mg mL^−1^). This indicates null cytotoxicity as the standard of non-hematotoxicity commonly refers to a hemolysis percentage below 5% [38].

In addition to this thiazolium molecule, PHEMA results from the hydrolysis of the cationic polymer. Nevertheless, PHEMA has been extensively analyzed and demonstrated good biocompatibility and no toxicity [33].

Therefore, the designed cationic polymer exhibits potent antibacterial activity against resistant strains while able to degrade in relatively short periods into well-defined and non-cytotoxic products, which is an important requisite for environmentally friendly materials and crucial in biomedical applications.

## 4. Conclusions

We have studied the degradation of a well-defined antibacterial polymer under different pH conditions leading to inactive products with low toxicity. The polymer was based on thiazolium groups attached on biocompatible PHEMA through carbonate pH-sensitive linkages. This polymer exhibited excellent antibacterial activity against resistant strain MRSA and relativity good hemotoxicity with HC50 value of 625 µg mL^−1^. The pH-induced degradation of the system analyzed by NMR demonstrated that the hydrolysis of the carbonate group occurs continuously, sustained over time, and faster at basic pH than at physiological pH. This hydrolysis left low cytotoxic and less effective products, reducing the risk of contamination and resistance development after the treatment.

## Data Availability

The data presented in this study are available on request from the corresponding author.
